# Exploring the active ingredients and potential mechanisms of action of sinomenium acutum in the treatment of rheumatoid arthritis based on systems biology and network pharmacology

**DOI:** 10.3389/fmolb.2023.1065171

**Published:** 2023-02-27

**Authors:** Nan Gong, Lin Wang, Lili An, YuanKun Xu

**Affiliations:** ^1^ Graduate School, Guizhou University of Traditional Chinese Medicine, Guiyang, China; ^2^ Orthopedic Surgery, First Affiliated Hospital of Guizhou University of Traditional Chinese Medicine, Guiyang, China; ^3^ Nephrology Department, National Clinical Research Center for Chinese Medicine Acupuncture and Moxibustion, First Teaching Hospital of Tianjin University of Traditional Chinese Medicine, Tianjin, China

**Keywords:** network pharmacology, sinomenium acutum, rheumatoid arthritis, target, molecular docking

## Abstract

**Objective:** To investigate and predict the targets and signaling pathways of sinomenium acutum (SA) in the treatment of rheumatoid arthritis (RA) through systems biology and network pharmacology, and to elucidate its possible mechanisms of action.

**Methods:** We screened the active ingredients and corresponding target proteins of SA in Traditional Chinese Medicine Systems Pharmacology Database and Analysis Platform (TCMSP), Traditional Chinese Medicines Integrated Database (TCMID) and Bioinformatics Analysis Tool for Molecular mechANism of Traditional Chinese Medicine (BATMAN); and obtained the targets of rheumatoid arthritis diseases in a database of gene-disease associations (DisGeNET), Online Mendelian Inheritance in Man (OMIM) database. The two targets were mapped by Venn diagram and the intersection was taken. The intersecting targets were used to construct protein-protein interaction (PPI) network maps in the String database, and Metascape was used for Gene Ontology (GO) functional annotation and Kyoto Encyclopedia of Genes and Genomes (KEGG) pathway enrichment. Finally, the molecular docking technique was applied to validate and further clarify the core target of SA for the treatment of rheumatoid arthritis.

**Results:** A total of six active ingredients and 217 potential targets were obtained after screening; 2,752 rheumatoid arthritis-related targets and 66 targets common to RA and SA. GO function and KEGG pathway enrichment analysis yielded 751 GO function entries (652 GO biological processes, 59 GO molecular functions and 40 GO cellular components) and 77 KEGG signaling pathways. It mainly involves pathways related to neural activity ligand-receptor interaction pathways, cancer pathways, calcium signaling channels, Th17 cell differentiation and others, which are mainly classified into four categories, including regulation of immunity, anti-inflammation, regulation of cell growth and apoptosis, and signaling. The molecular docking results showed that the binding energy of PTGS2, CASP3, JUN and PPARG to the key components beta-sitosterol, 16-epi-Isositsirikine, Sinomenine and Stepholidine were ≤ −6.5 kcal/mol, suggesting the existence of molecular binding sites.

**Conclusion:** SA acts on key targets such as PTGS2, CASP3, JUN, and PPARG to modulate signaling pathways such as neural activity ligand-receptor interaction, cancer, calcium ion, NF-κB, and Th17 cell differentiation to regulate immunity, anti-inflammation, modulation of cell cycle, bone metabolism, and signaling for the treatment of RA. It was also confirmed that the treatment of RA with SA has multi-component, multi-target, multi-pathway and multi-mechanism characteristics.

## 1 Introduction

Rheumatoid arthritis (RA) is a chronic, systemic, autoimmune syndrome. It is characterized by non-specific, symmetric inflammation of the peripheral joints, chronic inflammation and hyperplasia of the synovial membrane, formation of vascular opacities, invasion of articular cartilage, subchondral bone, ligaments and tendons, etc., resulting in destruction of articular cartilage, bone and joint capsule, ultimately leading to joint deformity and loss of function. Some patients are associated with varying degrees of systemic manifestations, and in severe cases, a variety of cardiovascular, pulmonary, and skeletal complications (Smolen et al., 2016; Xu et al., 2019; Scherer, Häupl, & Burmester, 2020). In the lungs, RA presents in different ways and may include airway, parenchymal, vascular and/or pleural disease. These include: interstitial lung disease, rheumatoid nodules, rheumatoid pneumoconiosis, etc. The most common form of vascular involvement in RA is rheumatoid vasculitis, which is characterized pathologically by the presence of destructive inflammatory infiltrates within the walls of small and medium-sized vessels (Yunt and Solomon, 2015). Rheumatoid arthritis is associated with an increased risk of cardiovascular events, such as myocardial infarction and stroke (Kitas and Gabriel, 2011). Epidemiological studies have shown that RA can occur at any age of development, and the ratio of women to men is about 3:1 (Quan, 2020). The current global prevalence of rheumatoid arthritis is approximately 1% (Radu and Bungau, 2021). However, RA is a major cause of disability and workforce loss Guidelines for Clinical Diagnosis and Treatment (2010). The disease not only affects the daily life activities of patients, but also imposes varying degrees of emotional and financial burdens on patients and their families. The pathogenesis of RA is still unclear, and it is speculated that the possible mechanisms are related to immune cells, inflammatory factors, matrix metalloproteinases, genetic and survival environmental factors. When an infection occurs in the body, immune cells cause inflammation and produce a large number of inflammatory cells, reactive oxygen species (ROS), and inflammatory factors, among which ROS can activate several signaling pathways downstream of RA disease and lead to joint damage. Cyclooxygenase-2 (COX-2) is a key enzyme for the synthesis of arachidonic acid (AA) by PGs and LTs. COX-2 can be abundantly expressed under the induction of inflammatory factors, thus contributing to the synthesis of PGs and exacerbating the inflammatory response. Inflammatory factors (e.g., TNF-α, IL-1β, etc.) simultaneously stimulate the production of MMPs, especially MMP-2, which in turn leads to irreversible damage to cartilage and bone. (Yamanaka et al., 2000; Litinsky et al., 2006; Yousefi et al., 2013; Hakim et al., 2018; Tokai et al., 2018; Singh et al., 2020). Among them, however, most studies consider the release of large amounts of inflammatory cytokines by T lymphocytes and macrophages causing osteoarthritic destruction as the main mechanism in the pathogenesis of RA. (Luque-Campos et al., 2019). Studies have identified more than one hundred loci associated with the risk of rheumatoid arthritis, most of which involve immune mechanisms, some of which are shared with other chronic inflammatory diseases. (Roberson and Bowcock, 2010). In the treatment of pain relief and reduction of inflammation, NSAIDS with Glucocorticoids are currently used as the first-line drugs. NSAIDS work as fast-acting drugs that block the synthesis of prostaglandins, prostacyclins and thromboxane by inhibiting cyclooxygenase, but are often associated with side effects such as nausea, abdominal pain, ulcers and gastrointestinal (GI) bleeding. Glucocorticoids are more effective anti-inflammatory drugs than NSAIDs in that they work by blocking the release of phospholipids and reducing the action of eosinophils, thereby reducing inflammation. And their side effects include osteoporosis, weight gain, diabetes, and immunosuppression. Anti-rheumatic drugs (DMARDs) are often used as second-line agents for rheumatoid arthritis because of their slower effectiveness. They are used to promote pain relief by slowing or stopping the progression of joint destruction and deformity and include drugs such as methotrexate (MTX), hydroxychloroqyine (Plaquenil) and sulfasalazine (Azulfidine) (Bullock et al., 2018).

Sinomenium acutum (SA) is the vine stem of Qing Feng Vine or Mao Qing Vine, etc., of the family Fangjiidae, mainly distributed in China and Japan. The use of SA was first recorded in Bencao Tujing, which has been used from ancient times to the present (Du et al., 2022). In China, Japan, and Korea, SA and sinomenine are used to treat neuralgia and rheumatoid arthritis with good reputation (Yamasaki, 1976). Modern pharmacological studies have found that SA has various pharmacological effects such as anti-inflammatory, analgesic and immunosuppressive effects (Yanqun, Xiaolan et al., 2022). Identification of anti-inflammatory components in SA by spectro-effective relationship and chemometric methods showed that sinomenine, magnoflorine, menisperine, cephalotenine were the main anti-inflammatory components of SA (Wang et al., 2019). Among them, SIN has a variety of pharmacological effects (Ansari et al., 2008; Zhou et al., 2008). It is a morphine derivative and is related to opioids such as levorphanol (Liu et al., 2006; Gupta et al., 2019). It has analgesic, anti-inflammatory, immunosuppressive, antihypertensive, and anti-arrhythmic activities, and is mainly used in the clinical treatment of rheumatoid arthritis (Liu et al., 1994; Wang et al., 2003; Yi et al., 2004; Gupta et al., 2019). It can also shorten the course of the disease and improve the prognosis of RA (Qiang et al., 2019). In extensive clinical trials, subcutaneous (s.c.) or per os (p.o.) administration of sinomenine has been very effective in relieving pain, swelling, and other symptoms of acute and chronic rheumatoid arthritis (Yamasaki, 1976). Its antirheumatic effects are thought to be mainly through mediating the release of histamine and inhibiting biological processes such as prostaglandin, leukotriene and nitric oxide synthesis (Liu et al., 2006). SA is receiving more attention from domestic and international researchers. Sinomenine related preparations such as Zhengqing Fengtong Ning (ZQFTN) tablets and injections, hydrochloride tablets and injections, and total alkaloids tablets and extended release Sinomenine preparations have been widely used in clinical practice (Xiangjin et al., 2019; Du et al., 2022). A meta-analysis (Liu et al., 2016)of 16 clinical trials involving 1,500 patients with RA showed that sinomenine showed better performance and fewer side effects compared with methotrexate (MTX) in the treatment of RA and better efficacy in combination with MTX. Another meta-analysis (Chen et al., 2015)of 956 patients with RA in 11 clinical studies also showed that ZQFTN combined with MTX was more effective than MTX alone in the treatment of RA and may have advantages over MTX in terms of adverse drug reactions. Min Xu et al. systematically evaluated the efficacy and safety of cytisine by systematically searching 43 electronic databases comparing sinomenine with non-steroidal anti-inflammatory drugs (NSAIDs). A total of 121 relevant studies were involved, with 10 quality-passed Jadad scales, involving a total of 1,185 patients with rheumatoid arthritis. The results showed that 1) with regard to chemical indicators: the rate of improvement and disappearance of rheumatoid factor was significantly higher in patients treated with sinomenine than in those treated with NSAIDs (*p* < 0.00001 and *p* = 0.008).2) In terms of clinical symptoms: compared with NSAIDs, sinomenine was more effective in improving morning stiffness (*p* < 0.00001), joint pain (*p* = 0.03) and erythrocyte sedimentation rate (*p* < 0.00001), but there was no significant difference between the two therapies in the treatment of joint swelling, grip strength and C-reactive protein (*p* > 0.05).3) Safety: the frequency of digestive adverse events was lower during sinomenine treatment than during NSAID treatment (*p* = 0.0003), while neurological adverse events were similar for both treatments (*p* = 0.31) (Xu et al., 2008). *In vivo* experiments showed that cyantoin ameliorated arthritis through MMPs, TIMPs and cytokines in rats (Zhou et al., 2008). *In vitro* experiments showed that cyantoin inhibited the inflammatory response of human fibroblast-like synoviocytes in rheumatoid arthritis through the TLR4/MyD88/NF-κB signaling pathway (Yao et al., 2017). Sinomenine is one of the main efficacy components of SA for the treatment of RA.

**FIGURE 1:**
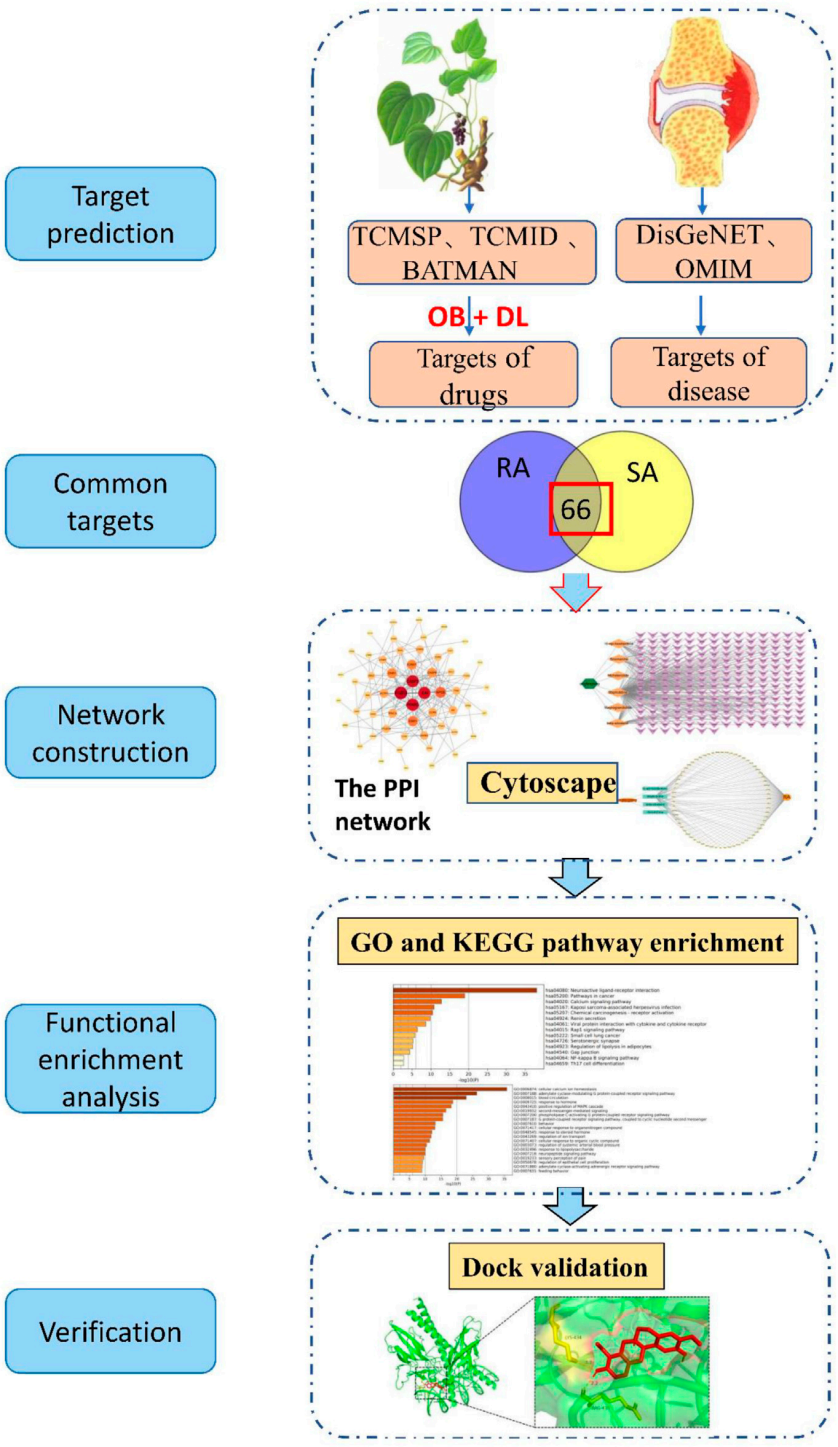
The workflow of the analysis for this study. TCMSP (Traditional Chinese Medicine Systems Pharmacology Database and Analysis Platform), TCMID (Traditional Chinese Medicines Integrated Database), BATAMN (Bioinformatics Analysis Tool for Molecular mechANism of Traditional Chinese Medicine), DisGeNET (a database of gene-disease associations), OMIM (Online Mendelian Inheritance in Man), OB (oral bioavailability), DL (drug-likeness), RA (Rheumatoid arthritis), SA (Sinomenium acutum), PPI (protein-protein interaction network), GO (Gene Ontology), KEGG (Kyoto Encyclopedia of Genes and Genomes).

The concept of network pharmacology was first proposed by Li et al., in 2014 (Li et al., 2014), and is a multidisciplinary integration based on systems biology theory. It has been widely used in recent years for the prediction of active ingredients and mechanisms of action of traditional Chinese medicines, and has provided a powerful aid for the development of new Chinese medicines (Chunwei et al., 2016; Dong et al., 2020; Ziyi et al., 2022). Currently, the first-line drugs for RA are mainly non-steroidal anti-inflammatory drugs (NSAIDs), but their side effects are more obvious. Traditional Chinese medicine has a long history in the treatment of RA, with significant efficacy and fewer side effects. In China, Japan and Korea, SA and sinomenine have a good reputation for the treatment of RA. In extensive clinical trials sinomenine preparations have also shown unique advantages. Sinomenine preparations is one of the main efficacious components of SA for the treatment of RA. Therefore, by tracing the mechanism of action of SA, the plant source of sinomenine, in the treatment of RA, this study was conducted to explore the possible mechanisms of action of Cymbopogon flexuosus in the treatment of RA by using network pharmacology and molecular docking techniques to provide scientific basis and guidance for clinical use. It is more beneficial to promote the development of new drugs. The workflow diagram of this study is shown in Figure 1.

## 2 Materials and methods

### 2.1 Acquisition of active ingredients and targets of SA and construction of network

Using the Chinese pinyin or Latin word of SA as the search term, the chemical composition of SA was searched in the Traditional Chinese Medicine Systems Pharmacology Database and Analysis Platform (TCMSP) and screened according to pharmacokinetic (ADME): oral bioavailability (OB) ≥ 30% and drug-like properties (DL) ≥ 0. 18. We obtained the active ingredients and corresponding target proteins of SA, and finally standardized the target gene names by the Uniprot database. The target protein supplementation of the active ingredients of Qingfengteng was done in the Traditional Chinese Medicines Integrated Database (TCMID) and Bioinformatics Analysis Tool for Molecular mechANism of Traditional Chinese Medicine (BATMAN). And the Uniprot database was also used to standardize the target gene names. The network diagram of the relationship between cyanobacteria-active ingredient-target was also constructed with the help of cytoscape 3.9.1.

### 2.2 Access to RA disease targets

The RA-related targets were retrieved from the database of gene-disease associations (DisGeNET) (https://www.disgenet.org/) and Online Mendelian Inheritance in Man (OMIM) (http://omim.org/) using “Rheumatoid Arthritis” as the search term. The targets were then converted into standard protein gene names in the UniProt database (https://www.uniprot.org/).

### 2.3 Prediction of potential targets for the treatment of RA with SA

The obtained gene names of SA and RA targets were uploaded to Venny online mapping tool (http://bioinfogp.cnb.csic.es/tools/venny/index.html) to obtain the intersection targets of cytosine and rheumatoid arthritis. And the drug-component-potential action target-disease network map was constructed by Cytoscape 3.9.1.

### 2.4 Protein-protein interaction network construction and key target screening

The obtained intersecting targets were used to construct protein-protein interaction (PPI) network maps in the String database (https://string-db.org). Select mutiple proteins, upload the intersection target genes, and set the corresponding parameters: the protein species is limited to “*Homo sapiens*”, the minimum interaction threshold is selected as “medium confidence (0.400)", and other parameters were kept as default settings to obtain the PPI network graph. The key targets were filtered out by degree greater than the mean value.

### 2.5 Gene Ontology (GO) enrichment analysis and Kyoto Encyclopedia of Genes and Genomes (KEGG) pathway enrichment analysis

To illustrate the role of potential targets of SA in gene functions and pathways, the obtained crossover targets of SA and RA were subjected to GO functional annotation and KEGG signaling pathway enrichment analysis by Metascape gene analysis software (https://metascape.org/gp/index.html). The species was selected as “*H. sapiens*”, and the default settings were Min Overlap of 3, *p* value < 0.01, and Min Enrichment of 1.5.

### 2.6 Primary active ingredient-target molecular docking

To validate the accuracy of the screened key targets of SA for RA, the active ingredients associated with potential targets of action screened by the regulatory network were molecularly docked to the top four target degree values of the core. Download the receptor (core target protein) 3D structure in the Protein Data Bank (PDB) database (https://www.pdbus.org). Download the compound structures of ligands (active ingredients of drugs) in the TCMSP database (TCMSP-raditional Chinese Medicine Systems Pharmacology Database and Analysis Platform (tcmsp-e.com). The receptors were processed by Pymol to remove water molecules, small molecule ligands, and hydrogenation, and then the ligand format was converted using AutoDock Tools 1.5.6 and the corresponding active pockets (Grid Box) were identified. Finally, molecular docking was performed using Autodock Vina software. Screening was performed based on binding energy < -6.5 kcal/mol, and the docking structures that met the conditions with the lowest binding energy were visualized with the help of Pymol.

## 3 Results

### 3.1 Active ingredients and potential targets of sinomenium acutum for the treatment of rheumatoid arthritis

**FIGURE 2:**
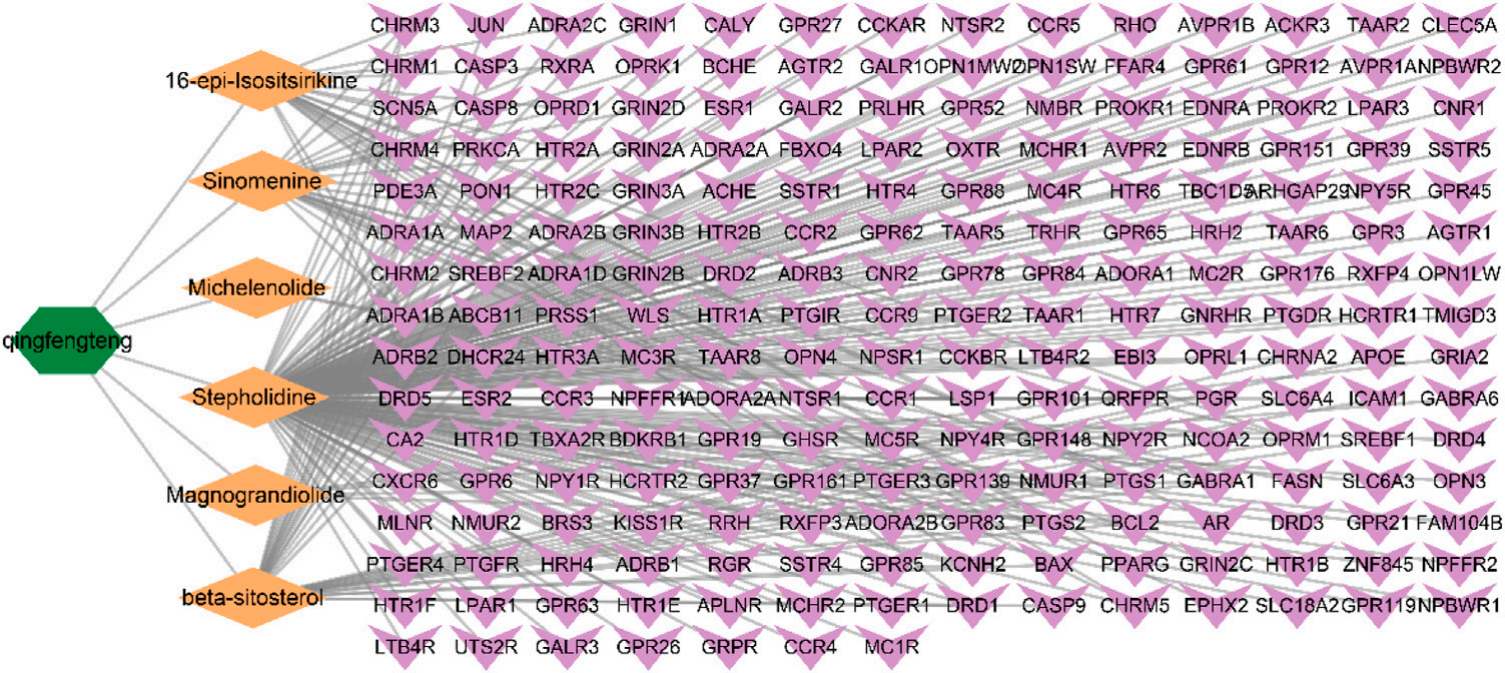
Network diagram of SA—Active ingredient—Target. The green color represents the drug SA, the orange diamond represents the active ingredient of SA, and the purple arrow represents the target protein. The lines represent the relationship between drug, active ingredient, and target.

The drug SA was searched through the TCMSP database, and six active ingredients of SA were obtained based on pharmacokinetic screening, namely, beta-sitosterol, 16-epi-Isositsirikine, magnograndiolide, michelenolide, sinomenine, stepholidine. The details are shown in Table 1. After de-weighting and obtaining activity corresponding to 45 targets. TCMID and BATMAN databases were supplemented with target proteins for the screened active ingredients of SA. Among them, 173 target proteins were screened by TCMID database and 41 target proteins were screened by BATMAN database. The target proteins from the above three major databases were combined, and 42 duplicate values were removed to obtain a total of 217 target proteins and their corresponding standard gene names. Construction of SA—Active ingredient—Target network map as shown in Figure 2.

**TABLE 1:** The active ingredients of SA.

MOL ID	Name	OB(%)	DL
MOL000358	beta-sitosterol	36.91	0.75
MOL000621	16-epi-Isositsirikine	49.52	0.59
MOL000622	Magnograndiolide	63.71	0.19
MOL000623	Michelenolide	47.54	0.25
MOL000625	Sinomenine	46.09	0.53
MOL000627	Stepholidine	33.11	0.54

### 3.2 RA target screening results

A total of 2,722 and 42 potential targets for rheumatoid arthritis were obtained from the DisGeNET and OMIM databases, respectively. A total of 2,752 disease-related targets were obtained after de-duplication and combination of the two targets.

### 3.3 Potential target of SA in treating RA

**FIGURE 3:**
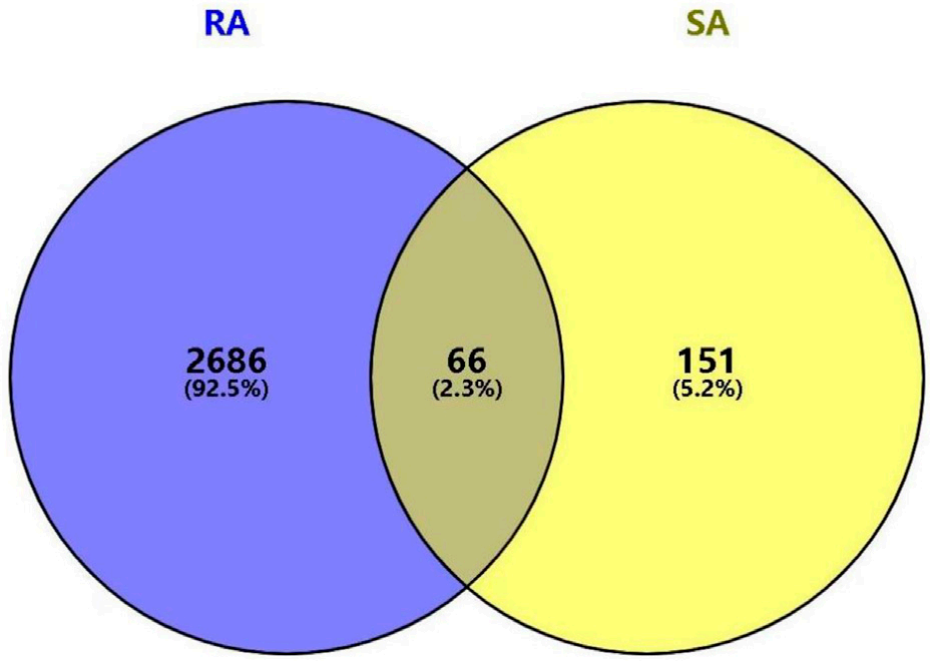
Venn diagram of the intersection target of SA and RA.

**FIGURE 4:**
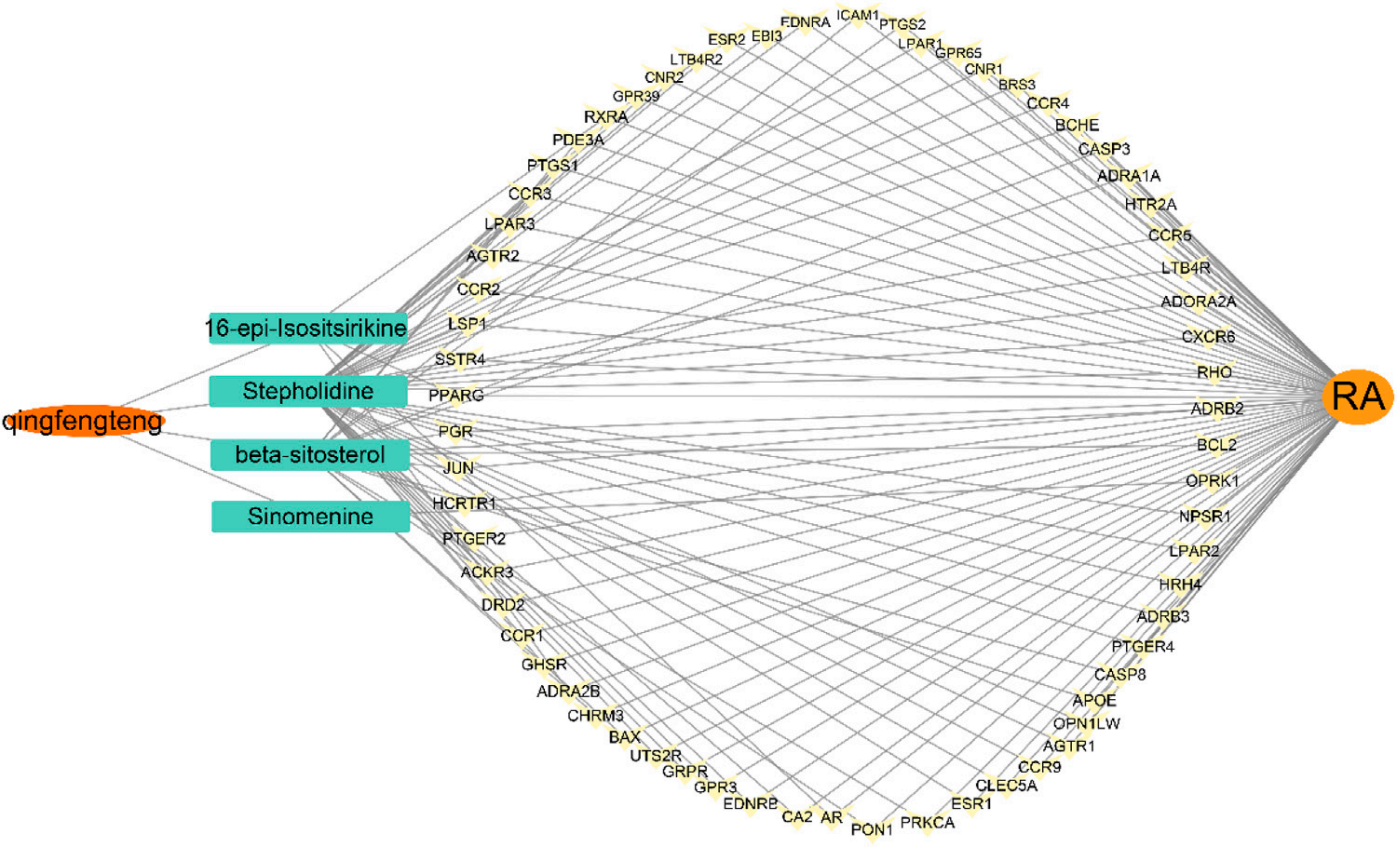
Network diagram of SA-Components-Potential targets of action-RA. The orange oval represents the drug (SA), the orange circle represents RA, the middle cyan rectangle represents the active ingredient, and the yellow represents the potential target of action between the two. The lines represent the relationship between SA, active ingredient, potential target of action, and RA.

Mapping the gene names of drug targets and disease targets obtained in 2.1 and 2.2 to each other by Venn diagram, and 66 intersecting targets for SA and RA were obtained, as shown in Figure 3. A SA—Components—Potential targets of action—RA network diagram was constructed by Cytoscape 3.9.1, as shown in Figure 4.

### 3.4 Results of PPI network construction and core target screening

**FIGURE 5:**
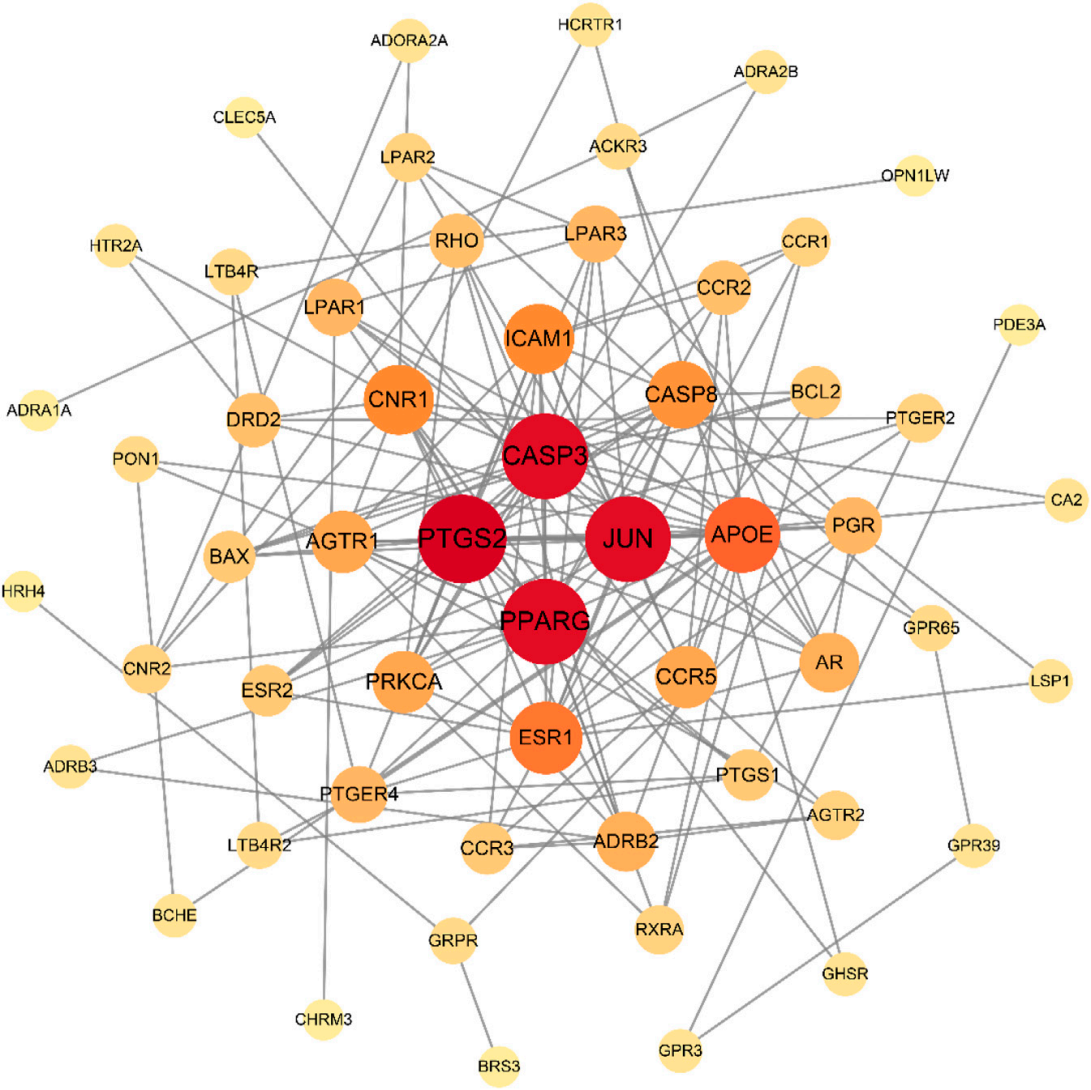
PPI network of SA for RA. A total of 56 nodes and 160 lines are included. The larger the node, the redder the color, the larger the degree. The more lines between nodes, the larger the between centrality. The larger the degree and between centrality, the more important the node is.

**FIGURE 6:**
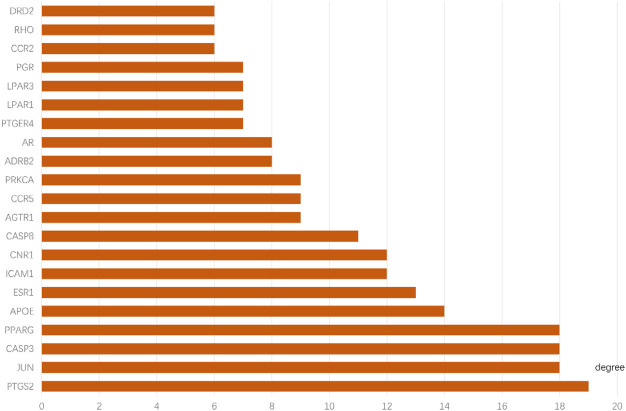
Key targets for the treatment of RA with SA. the y-axis displays significant top 21 genes, and the x-axis shows degree counts of these genes.

The 66 intersecting targets obtained were used to construct protein-protein interaction (PPI) network maps in the String database, and 10 target genes without interaction were excluded, for a total of 56 genes to construct network maps, as shown in Figure 5. There were 21 genes with degree values greater than the mean value, as shown in Figure 6. Sorting degree from high to low, the top 4 were PTGS2 (degree = 19), CASP3 (degree = 18), JUN (degree = 18), and PPARG (degree = 18) in that order. It indicates that four protein genes, PTGS2, CASP3, JUN, and PPARG, are more likely to be involved in the treatment of RA by SA.

### 3.5 GO functional enrichment and KEGG pathway enrichment analysis

**FIGURE 7:**
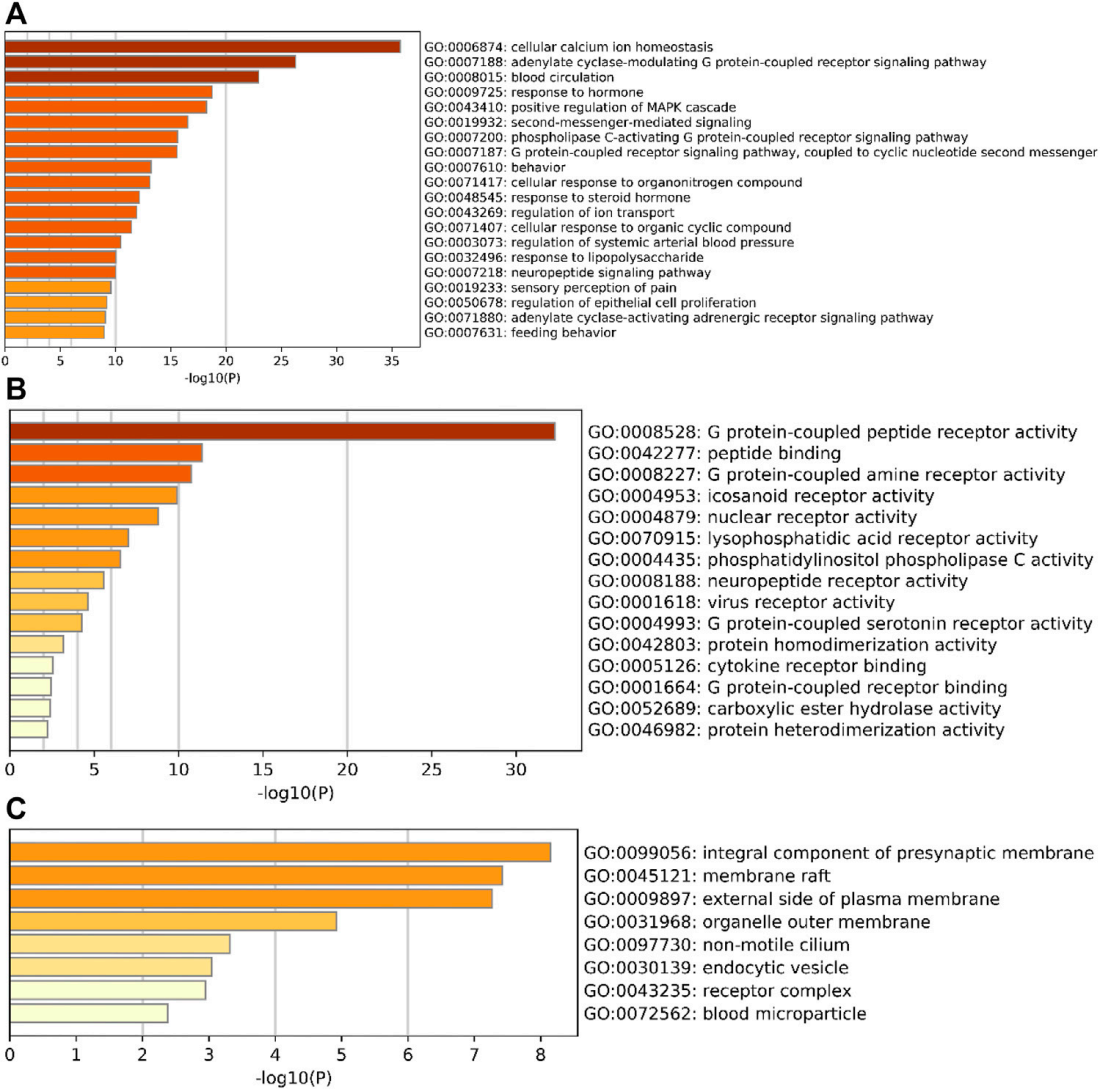
Bar graph of GO gene function annotation. **(A)** BP, **(B)** MF; **(C)** CC. horizontal coordinates represent *p*-values taken at log base 10, vertical coordinates represent GO entry names, and colors from light to dark represent the degree of significance.

The potential action targets of SA for RA treatment were entered into metascape gene analysis software for GO gene annotation and KEGG pathway enrichment analysis. The results showed that there were 751 GO gene annotation entries. Among them, there are 652 entries of biological process (BP), mainly involving cellular calcium ion homeostasis, adenylate cyclase-modulating G protein-coupled receptor signaling pathway, blood circulation, etc. A total of 59 entries were enriched for molecular function (MF), mainly related to G protein-coupled peptide receptor activity, peptide binding, G protein-coupled amine receptor activity, etc. A total of 40 entries were enriched for cellular composition (CC), mainly involving the integral component of presynaptic membrane, membrane raft, and external side of plasma membrane, etc. See Figures 7A–C for details.

**FIGURE 8:**
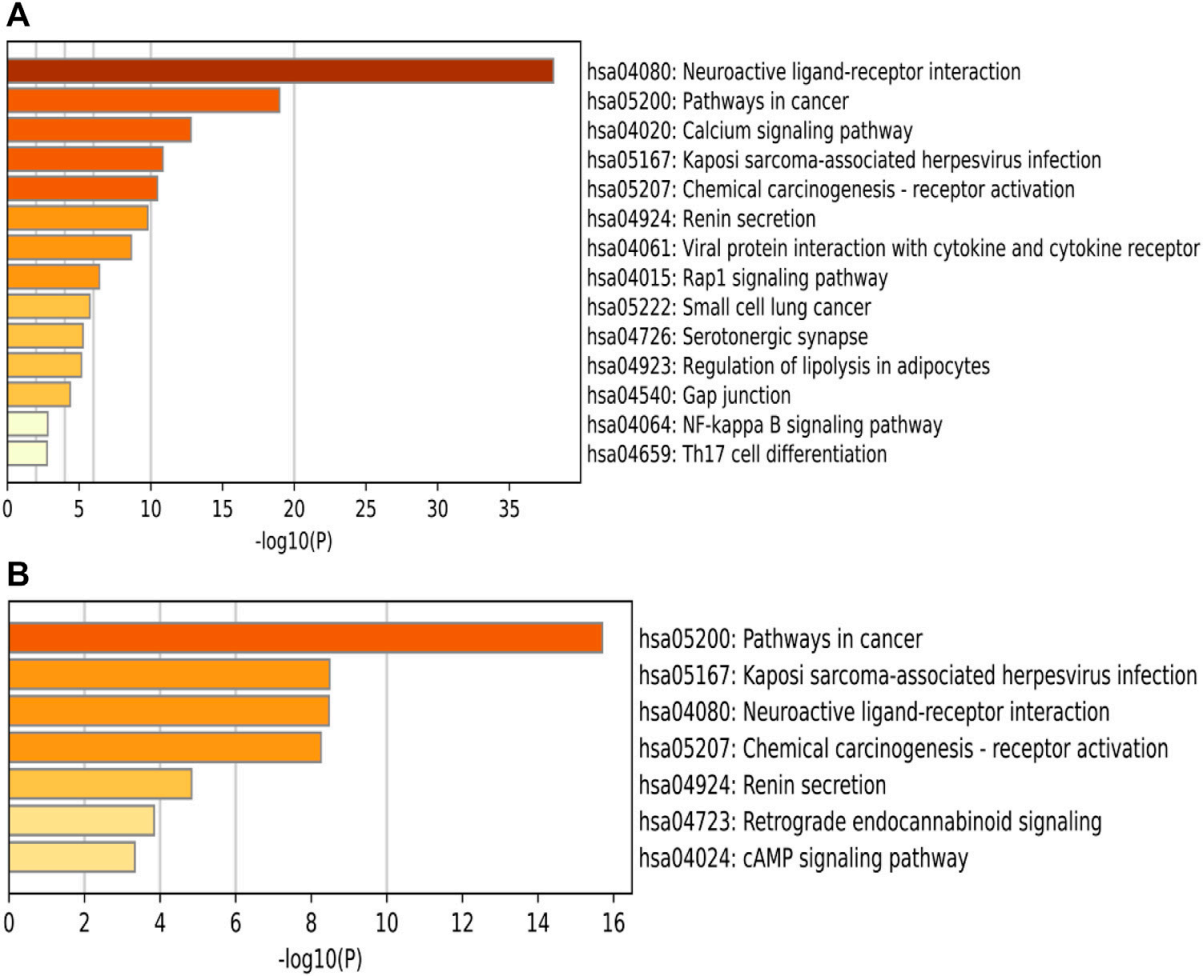
Bar graph of KEGG enrichment pathways. The horizontal coordinates represent the *p*-values with log base 10 and the vertical coordinates represent the pathway names. The color represents the enrichment significance, the darker the color, the higher the enrichment. Figure **(A)** show KEGG PATHWAY information *via* intersecting targets, Figure **(B)** show KEGG PATHWAY information *via* key targets.

A total of 77 pathways were enriched by KEGG, and the log(10)*p* values were sorted from smallest to largest, and the top 14 pathways with the most significant enrichment were selected and plotted in a bar graph, see Figures 8A, B shows KEGG pathway information *via* key targets. It shows 44 pathways associated with the 21 key targets. Excluding the less relevant pathways, the top three pathways were Neuroactive ligand-receptor interaction, Pathways in cancer, and Calcium signaling pathway, in order of ranking. Classification of the screened pathways yielded that SA may play a therapeutic role in RA by reference to four aspects: immune modulation, anti-inflammation, cell cycle regulation and signaling (Table 2). The classification of signaling pathways in Table 2 is referenced from the KEGG database, which provides a specific classification of pathway attribution.

**TABLE 2:** Breakdown of pathway classification related to RA treatment by SA.

Class	Signaling pathway
Immune System	Neuroactive ligand-receptor interaction、Pathways in cancer、IL-17 signaling pathway、C-type lectin receptor signaling pathway、Natural killer cell mediated cytotoxicity、NOD-like receptor signaling pathway、Chemokine signaling pathway、NF-kappa B signaling pathway、Th17 cell differentiation
Signal transduction	Calcium signaling pathway、TNF signaling pathway、cGMP-PKG signaling pathway、cAMP signaling pathway、Viral protein interaction with cytokine and cytokine receptor、Cytokine-cytokine receptor interaction、Rap1 signaling pathway、Phospholipase D signaling pathway、PI3K-Akt signaling pathway、Sphingolipid signaling pathway
Cellular Processes; Cell growth and death	Apoptosis—multiple species、Apoptosis、p53 signaling pathway、Necroptosis
Inflammation	Inflammatory mediator regulation of TRP channels

^a^ Classification of the screened pathways according to the KEGG, database yielded that SA may play a therapeutic role in RA, by reference to four aspects: immune modulation, anti-inflammation, cell cycle regulation and signaling.

### 3.6 Molecular docking results

**FIGURE 9:**
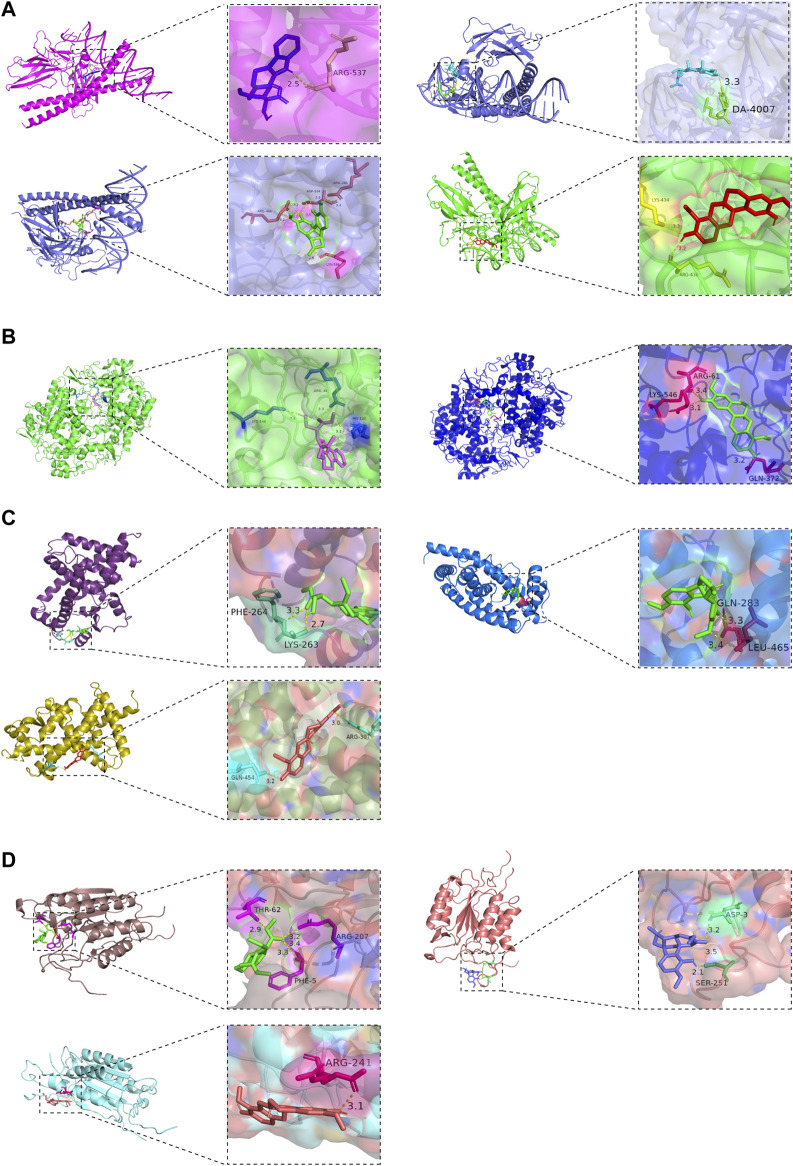
Molecular docking of the active ingredients of SA with key targets. **(A)** JUN target is hydrogen-bonded to the active ingredient of SA; **(B)** PTGS2 target is hydrogen-bonded to the active ingredient of SA; **(C)** PPARG target is hydrogen-bonded to the active ingredient of SA; **(D)** CASP3 target is hydrogen-bonded to the active ingredient of SA.

The molecular docking results showed that the active ingredients beta-sitosterol, 16-epi-Isositsirikine, sinomenine and stepholidine have binding sites with PTGS2, CASP3, JUN and PPARG. Their corresponding minimum binding energies were all below −6.5 kcal/mol (Table 3). It is suggested that intermolecular binding forces such as hydrogen bonding connection, π-π conjugation and hydrophobic stacking may exist between the active ingredient and the target. The hydrogen bonding connection between the active ingredient and the key target is shown in Figures 9A–D.

**TABLE 3:** Minimum binding energy between the active ingredients and the main targets of SA.

Active ingredients	Minimum binding energy/(kcal·mol^−1^)			
	PTGS2	CASP3	JUN	PPARG
beta-sitosterol	−8.0	−7.9	−8.2	−7.7
16-epi-Isositsirikine	−7.7	−6.6	−6.9	−7.2
Sinomenine	−8.1	−6.5	−7.5	−6.6
Stepholidine	−7.8	−7.0	−8.0	−6.6

## 4 Discussion

RA is designated as a refractory disease by the World Health Organization (Miao et al., 2017), and its etiology and pathogenesis are still unclear. RA is now thought to be closely related to a variety of factors including immune cells, inflammatory factors, matrix metalloproteinases, abnormal oxidative stress, genetic and survival environmental factors (Yamanaka et al., 2000; Litinsky et al., 2006; Yousefi et al., 2013; Hakim et al., 2018; Tokai et al., 2018; Singh et al., 2020). SA has been used in the treatment of RA for over 1,000 years (Yamasaki, 1976; Zhao et al., 2012; Gupta et al., 2019). Compared with methotrexate treatment, SA and its preparations have better clinical efficacy and fewer adverse events in the treatment of RA (Liu et al., 2016). The results of the present study indicate that the therapeutic effects of SA on RA are mainly achieved through immune modulation, anti-inflammation, regulation of bone cell metabolism, cell cycle and signaling, which is consistent with the currently accepted pathogenesis of rheumatoid arthritis and further supports the scientific nature of the mechanism of action of SA in the treatment of RA.

From the Network diagram of SA-Components-Potential targets of action-RA, it can be seen that the main components of SA for RA treatment are beta-sitosterol, 16-epi-Isositsirikine, sinomenine and stepholidine. It was shown that beta-sitosterol significantly reduced the levels of TNF-α, C-reactive protein, and IL-2 in the serum and tissues of rats with adjuvant arthritis (Kripa et al., 2011). And inhibits M1 macrophage polarization and enhances M2 macrophage polarization, thereby reducing symptoms in adjuvant arthritic mice (Liu et al., 2019). Also β-sitosterol modulates macrophage function and reduces rheumatoid inflammation in CIA mice (Liu et al., 2019). Sinomenine has been approved by the Chinese Food and Drug Administration (CFDA) for the treatment of RA (Liu et al., 2018). Sinomenine can reduce inflammatory cytokines *in vivo* and *in vitro* (Xiong and Yang, 2012; Xiong et al., 2017). In the rat arachidonic acid (AA) model, the levels of IL-1 and TNF-α pro-inflammatory factors in serum and joint fluids were reduced, and the levels of anti-inflammatory factors such as IL-4 and IL-10 were modulated, which significantly inhibited the secondary lesions of adjuvant arthritis in rats (Desen et al., 2006). Sinomenine also has an inhibitory effect on T and B cell activation (Jihong et al., 2005); it also promotes apoptosis of RA-fibroblast-like synoviocytes (FLS) by upregulating the expression of micro RNA-23b-3p and fibroblast growth factor 9 in RA fibroblast-like synoviocytes (Jia et al., 2020). In addition, sinomenine can also inhibit bone destruction by stimulating osteoclast (OC) to increase OPG secretion and decrease RANKL secretion, promoting osteoblast differentiation and maturation (Yang et al., 2018). Academician Guozhang Jin confirmed the dual pharmacological effects of stepholidine with partial agonism of dopamine D1 receptors and blockade of dopamine D2 receptors (Dalton et al., 2014; Peña et al., 2014). The pharmacology of 16-epi-Isositsirikine is poorly studied and needs further research in the future.

Analysis of the key target network map of PPI and SA for RA revealed that the top four targets with the highest degree of SA for RA were PTGS2, JUN, CASP3, and PPARG. Treatment with PTGS2 has been shown to reduce inflammation in rheumatoid arthritis rats by reducing serum levels of interleukin 1β, interleukin six and tumor necrosis factor α and their mRNA expression (Wei et al., 2020). JUN regulates the clinical course of rheumatoid arthritis by modulating the expression of pro-inflammatory cytokines and chemokines and inducing elevated Fre-1 expression and Fra-1 protein in peripheral blood in synovial macrophages (Hannemann et al., 2019). CASP3 is an apoptotic factor (Okamoto et al., 2000), and studies (Wu et al., 2019) have shown that CASP3 is involved in the cell scorching pathway, and that apoptosis and scorching are different ways of programmed necrosis of synovial fibroblasts, ultimately leading to changes in the number of abnormally proliferating synovial fibroblasts and the release of inflammatory factors, which affect the RA process. PPARG can enhance insulin sensitivity, promote adipocyte differentiation and adipogenesis by regulating the expression of related genes, and has anti-atherosclerotic, anti-inflammatory, and antioxidant properties (Huirun et al., 2020). There are fewer studies on PPARG in RA, which may be treated by its anti-inflammatory effects.

**FIGURE 10:**
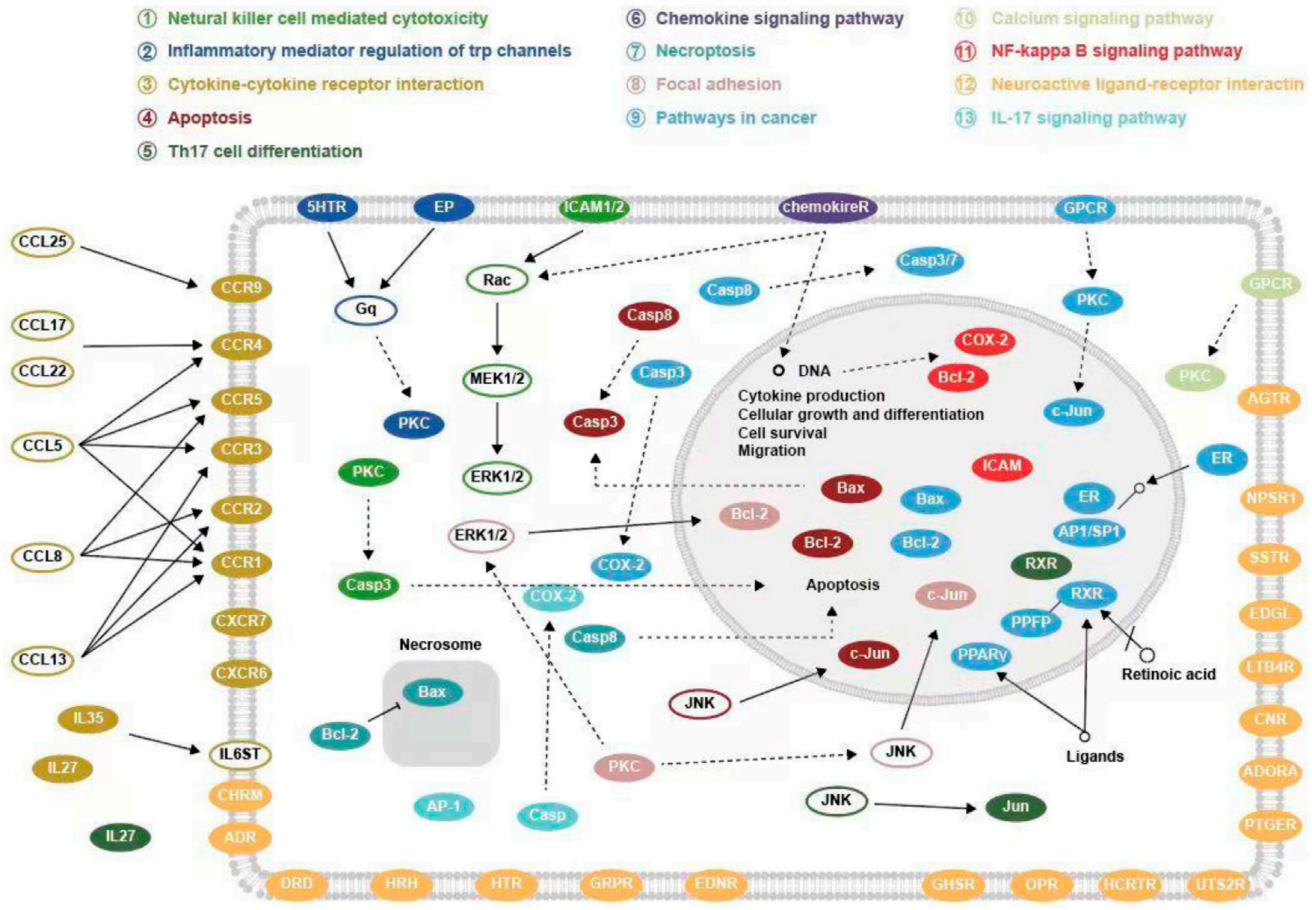
Possible targets and signaling pathways for the treatment of RA with SA.

GO functional enrichment analysis revealed that SA regulates biological processes (BP) such as G protein-coupled receptor signaling pathway and blood circulation through cellular calcium homeostasis, adenylate cyclase; molecular functions (MF)such as G protein-coupled peptide receptor activity, peptide binding, and G protein-coupled amine receptor activity; and cellular composition (CC) such as components of the presynaptic membrane, membrane rafts, and the outer side of the plasma membrane for RA. The results of KEGG pathway enrichment analysis showed that SA may treat RA by acting on signaling pathways such as Neuroactive ligand-receptor interaction, Pathways in cancer, Calcium signaling pathway, NF-kappa B signaling pathway, Th17 cell differentiation. Li et al. (Li et al., 2007) found that RA genes were significantly enriched in the neuroactive ligand-receptor interaction pathway. Studies have shown that RA is associated with an increased risk of cancer (Elandt and Aletaha, 2011). The abnormal proliferation of fibroblast-like cells in RA exhibits carcinoid features (Yoo et al., 2015). Calcium signaling pathway are essential for a variety of cellular functions, including differentiation, effector function and gene transcription in the immune system. Davies and Hallett (Davies and Hallett, 1998) found that calcium ion signaling in the cytoplasm can trigger neutrophil responses in patients with rheumatoid arthritis. IL-17 and NF-κB pathways are closely related to RA. IL-17 is the most important inflammatory cytokine produced by Th17. Metalloproteinases (MMPS), nitric oxide and RANK/RANKL receptor activators, can be upregulated by IL-17 in both cartilage and osteoblasts, leading to damage of bone and articular cartilage and promoting the development of RA (Lundy et al., 2007). Benito et al. (Benito et al., 2004)] found that synovial tissue from patients with early inflammatory arthritis showed high local NF-κB expression and played an important role in joint destruction. The possible targets and signaling pathways of SA for the treatment of RA are shown in Figure 10.

The molecular docking results showed that the active ingredients beta-sitosterol, 16-epi-Isositsirikine, sinomenine and stepholidine of SA bind well to the key targets PTGS2, JUN, CASP3, and PPARG had good binding performance with the lowest binding energy of −8.1 to −6.5 kcal/mol, and reached steady state quickly. It indicates that SA can be useful in the treatment of RA.

## 5 Conclusion

In summary, beta-sitosterol, 16-epi-Isositsirikine, sinomenine, and stepholidine are the main substance bases for the treatment of RA in SA, and PTGS2, JUN, CASP3, and PPARG are the potential targets of action. SA mainly treats RA by modulating inflammatory cytokines, immune cells, regulating cell cycle, such as apoptosis and scorching, and regulating osteoblasts and osteoclasts. This study also revealed the multi-functional synergistic mechanism of SA, involving the conduction pathways of Neuroactive ligand-receptor interaction, Pathways in cancer, Calcium signaling pathway, NF-kappa B signaling pathway, Th17 cell differentiation, further confirming the multi-component, multi-target, multi-pathway, and multi-mechanism characteristics of SA for the treatment of RA. In this study, we investigated the mechanism of action of SA, the source of sinomenine, in the treatment of rheumatoid arthritis by tracing the source, which is more beneficial to promote the development of new drugs. To provide a reference for in-depth research on the treatment of RA with SA. The shortcoming of this study is the prediction of the possible mechanism of action of SA in the treatment of RA by biological database and computer simulation, which needs to be verified by further basic research in the future.

## Data Availability

The original contributions presented in the study are included in the article/Supplementary Materials, further inquiries can be directed to the corresponding author.
