# Supplementary material for: Exploring the active ingredients and potential mechanisms of action of sinomenium acutum in the treatment of rheumatoid arthritis based on systems biology and network pharmacology
Source: Front Mol Biosci. 2023 Feb 27;10:1065171. doi: 10.3389/fmolb.2023.1065171 (PMC10009275; doi:10.3389/fmolb.2023.1065171)
Supplement: Supplementary file 3 [file Table1.DOCX]

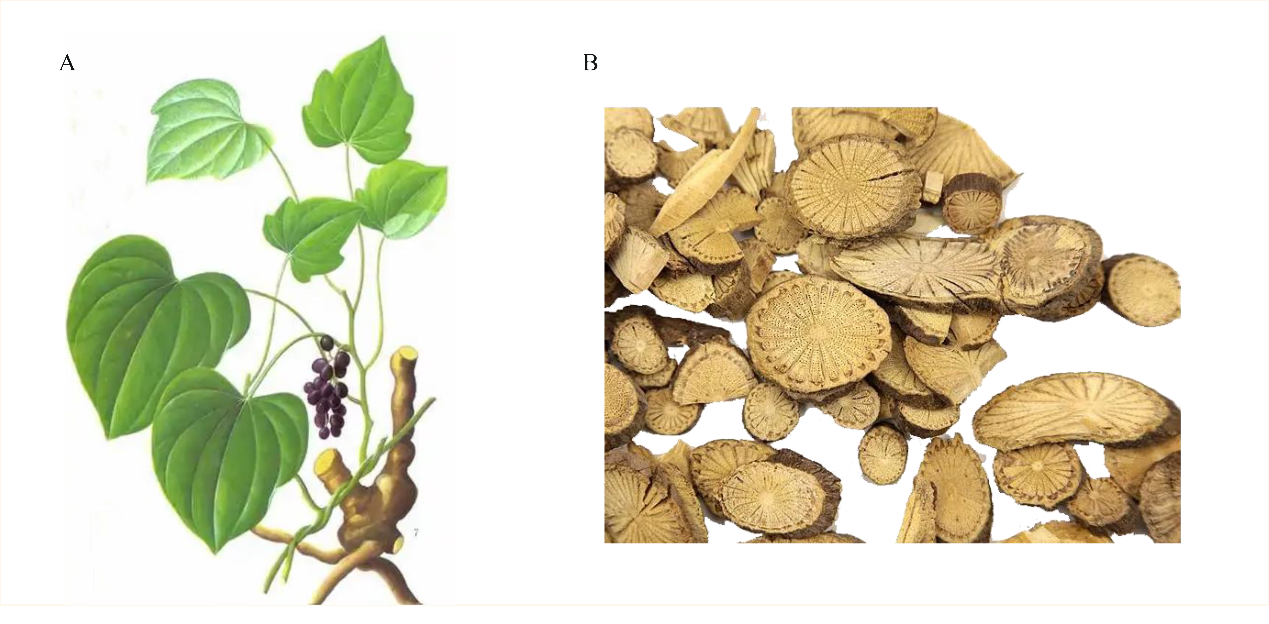


**Figure 1** Qing Feng Teng (*Caulis Sinomenii*). (A) Sinomenium acutum (Thunb.) Rehd. et Wils. var. cinereum Rehd.et Wils.；(B) *Caulis Sinomenii*.
